# Coupled dynamics of behaviour and disease contagion among antagonistic groups

**DOI:** 10.1017/ehs.2021.22

**Published:** 2021-03-18

**Authors:** Paul E. Smaldino, James Holland Jones

**Affiliations:** 1 University of California Merced, Merced, California, USA; 2 Stanford University, Palo Alto, California, USA

**Keywords:** Transmission dynamics, coupled contagion, homophily, outgroup aversion, social distancing

## Abstract

Disease transmission and behaviour change are both fundamentally social phenomena. Behaviour change can have profound consequences for disease transmission, and epidemic conditions can favour the more rapid adoption of behavioural innovations. We analyse a simple model of coupled behaviour change and infection in a structured population characterised by homophily and outgroup aversion. Outgroup aversion slows the rate of adoption and can lead to lower rates of adoption in the later-adopting group or even behavioural divergence between groups when outgroup aversion exceeds positive ingroup influence. When disease dynamics are coupled to the behaviour-adoption model, a wide variety of outcomes are possible. Homophily can either increase or decrease the final size of the epidemic depending on its relative strength in the two groups and on *R*_0_ for the infection. For example, if the first group is homophilous and the second is not, the second group will have a larger epidemic. Homophily and outgroup aversion can also produce dynamics suggestive of a ‘second wave’ in the first group that follows the peak of the epidemic in the second group. Our simple model reveals dynamics that are suggestive of the processes currently observed under pandemic conditions in culturally and/or politically polarised populations such as the USA.

## Graphical abstract

**Figure:**
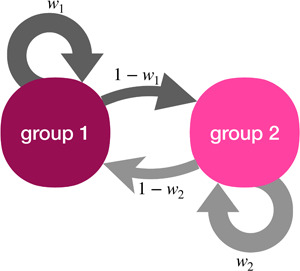

**Social media summary:** We modelled the joint spread of disease and preventive behaviour in a polarised population. Polarisation doesn't help.

## 1. Introduction

Behaviour can spread through communication and social learning like an infection through a community (Bass, 1969; Centola, 2018). Cavalli-Sforza and Feldman, who pioneered treating cultural transmission in an analogous manner to genetic transmission, noted that ‘another biological model may offer a more satisfactory interpretation of the diffusion of innovations. The model is that of an epidemic’ (Cavalli-Sforza & Feldman, 1981: 32–33). The biological success of *Homo sapiens* has been attributed to its capacity for cumulative culture, and particularly to the rapid and flexible adaptability that arises from social learning (Henrich, 2015). Adoption of adaptive behaviours during an epidemic of an infectious disease could be highly beneficial to both individuals and the population in which they are embedded (Fenichel et al., 2011). Coupling models of behavioural adoption and the transmission of infectious disease, what we call *coupled contagion* models, may thus provide important insights for understanding the dynamics and control of epidemics. While we might expect strong selection – both biological and cultural – for adaptive responses to epidemics, complications such as the potentially differing time scales of culture and disease transmission and the existence of social structures that shape adoption may complicate convergence to adaptive behavioural solutions.

In this paper, we explore the joint role of *homophily* – the tendency to form ties with people similar to oneself – and *outgroup aversion* – the tendency to avoid behaviours preferentially associated with an outgroup. Identity exerts a powerful force on the dynamics of behaviour (Hogg & Abrams, 2007; Bishop, 2009; Mason, 2018; Smaldino, 2019; Klein, 2020; Moya et al., 2020). This is because identity at least partly determines who we associate with, communicate with, and strive to either emulate or avoid. Our analysis is predicated on the idea that this matters for the dynamics of infection. For example, Salathé and Bonhoeffer (2008) showed that if rates of vaccine adherence cluster on networks, as when communities collectively adopt identity-based positions on the likely costs and benefits of vaccination (Bauch & Earn, 2004) or when like-minded individuals tend to assort in social networks (Bishop, 2009), the overall vaccination rates needed for herd immunity can be substantially higher than suggested by models that assume random vaccination.

Homophily involves interactions with ingroup members at rates higher than expected by chance. Homophily is often treated as though it were a global propensity for assortment by type (e.g. Centola, 2011). However, homophily is frequently observed to a greater or lesser degree across subgroups, a phenomenon known as differential homophily (Morris, 1991). Consider a case of two interacting groups, where one is more homophilous than the other. The less homophilous group may consist of more ‘frontline’ workers, who are exposed to a broader cross-section of the population by the nature of their work. In such cases, differential homophily may lead to differential disease dynamics in each group.

Members of opposed identity groups not only engage with the world differently, but they can react in divergent ways to identical stimuli. Asked to watch political debates or hear political arguments, partisans often grow more strongly partisan, to the consternation of moderates (Taber et al., 2009). In the USA, partisan identities have become increasingly defined in terms of their opposition to the opposing party (Abramowitz and Webster, 2016). When considering the adoption of products, consumers often become disenchanted with otherwise attractive purchases if the products are associated with identity groups viewed as different from their own (Berger & Heath, 2007, 2008). Smaldino et al. (2017) modelled the spread of a behaviour among members of two groups who responded positively to the behavioural contagion but tended to reject it if it was overly associated with the outgroup. They showed that outgroup aversion not only decreased the overall rate of adoption, but could also delay or even entirely suppress adoption in one of the groups. While populations vary in the extent to which they are polarised or parochial, identity clearly matters to the adoption of health behaviours in at least communities. For example, in the USA, people who identify with the right-wing Republican party are much less likely than those identifying with the centre-left Democratic party to endorse mask-wearing or belief in its efficacy in preventing disease transmission during the COVID-19 pandemic (van Kessel & Quinn, 2020).

Several previous studies have considered the coupled contagion of behaviour and infection, usually focused on cases where the behaviour is one that decreases the spread of the disease (such as social distancing or wearing face masks) and sometimes using the assumption that increased disease prevalence promotes the spread of the behaviour (Tanaka et al., 2002; Epstein et al., 2008; Funk et al., 2010; Verelst et al., 2016; Fast et al., 2015; Fu et al., 2017; Hébert-Dufresne et al., 2020; Mehta & Rosenberg, 2020). These models typically assume that individuals differ only in behaviour and disease status. Thus, the spread of both disease and behaviour depend primarily on rates of behaviour transmission and disease recovery. This is true even of models in which the population is structured on networks. Network structure can change the dynamics of contagion. However, contrary to the assumptions of most models, behavioural distributions on social networks are anything but random. People assort in highly non-random ways (McPherson et al., 2001) and these non-random associations both drive and are driven by social identity. This suggests that the role of social identity is an important, but under-studied, component of coupled contagion models.

Here, we consider how identity – and particularly homophilous interactions with ingroup members and aversion to adopt behaviours used by an outgroup – influences the spread of novel behaviours that consequently affect the transmission of infectious disease. The model we will present is complex, and hence challenging to analyse. To help us make sense of the dynamics, we will first describe the dynamics of infection and behaviour adoption in isolation, and then explore the full coupled model. We will first show how homophily can introduce temporal delays in the infection trajectories between groups. We will then show how outgroup aversion can lead to reduced or even fully inhibited behaviour adoption by the later-adopting group. Finally, we will analyse the fully coupled model and show how the identity-driven forces we consider can lead differentiated identity groups to experience an epidemic in very different ways.

## 2. The SIR model of infection with homophily

We model infection in a population in which individuals can be in one of three states: susceptible, infected and recovered. When susceptibles interact with infected individuals, they become infected with a rate equal to the effective transmissibility of the disease, *τ*. Infected individuals recover with a constant probability *ρ* per infection per unit time. This is the well-known SIR model of epidemics (Kermack & McKendrick, 1927; Tolles & Luong, 2020). The baseline model assumes random interactions governed by mass action, and the dynamics are described by well-known differential equations. This model yields the classic dynamics in which the susceptible and recovered populations appear as nearly mirrored sigmoids, while the rate of infected individuals rises and falls. The threshold for the epidemic is given by the basic reproduction number, *R*_0_, which is a measure of the expected number of secondary cases caused by a single, typical primary case at the outset of an epidemic in a population entirely composed of uninfected individuals. An epidemic occurs when *R*_0_ > 1. For the basic SIR model in a closed population, *R*_0_ = *τ*/*ρ*.

Our analysis will focus on scenarios where individuals assort based on identity. In this case, assume that individuals all belong to one of two identity groups, indicated with the subscript 1 or 2. Let *w_i_* be the probability that interactions are with one's ingroup, *i* ϵ {1,2}. It is therefore a measure of homophily; populations are homophilous when *w_i_* > 0.5. It is important to recognise that groups can differ in their homophily (Morris, 1991). For example, if groups differ in socioeconomic class and group 1 tends to employ members of a group 2 as service workers, homophily will be higher for group 1; a member of group 2 is more likely to encounter members of group 1 than the reverse. We can update the equations governing infection dynamics for members of group 1, with analogous equations governing members of group 2.
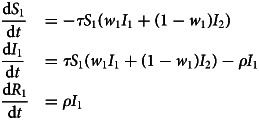


**Figure 1.**
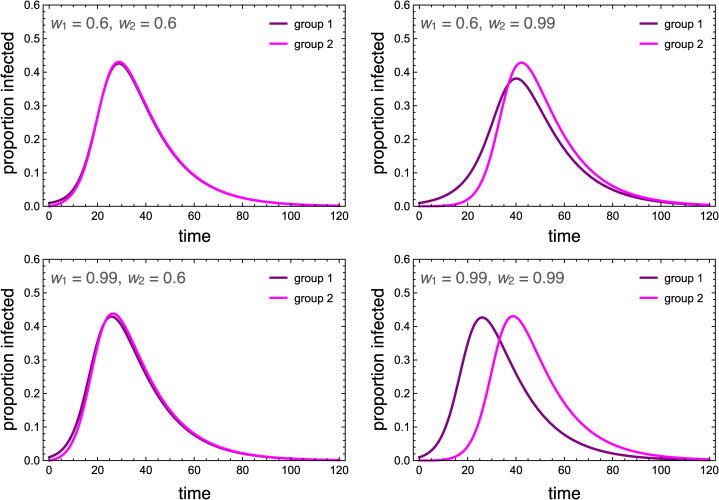
Dynamics of the infected population of each group under low and high homophily (*w_i_* = 0.6, 0.99). Other parameters used were *τ* = 0.3, *ρ* = 0.07, *I*_1_(0) = 0.01, *I*_2_(0) = 0. *R*_0_ ≈ 4.28 in the absence of homophily.

We assume the disease breaks out in one of the two groups, so the initial number infected in group 1 is small but non-zero, while the initial number infected in group 2 is exactly zero. Without loss of generality, we have assumed that group 1 is always infected first. When homophily is low, the model exhibits standard SIR dynamics approximating a single unified population. When an infection breaks out in group 1, homophily can delay the outbreak of the epidemic in group 2. Homophily for each group works somewhat synergistically, but the effect is dominated by *w*_2_. This is because the infection spreads rapidly in a homophilous group 1, and if group 2 is not homophilous, its members will rapidly become infected. However, if group 2 is homophilous, its members can avoid the infection for longer, particularly when group 1 is also homophilous. If only group 2 is homophilous, the initial outbreak will be delayed, but the peak infection rate in group 2 can actually be higher than in group 1, as the infection is driven by interactions with both populations (Figure 1).

We also considered the case in which the transmissibility of the infection can be reduced to very near the recovery rate, so that *R*_0_ is very close to 1. In this case, homophily can protect groups where infection did not originally break out by keeping members relatively separated from the infection group (Figure S2).

## 3. Behavioural contagion with outgroup aversion

We model behaviour adoption as a susceptible–infectious–susceptible (SIS) process, in which individuals can oscillate between adoption and non-adoption of the behaviour indefinitely. We view this as more realistic than an SIR process for preventative-but-transient behaviours like social distancing or wearing face masks. To avoid confusion with infection status, we denote individuals who adopted the preventative behaviour as careful (*C*), and those who have not as uncareful (*U*). Unlike a disease, which is reasonably modelled as equally transmissible between any susceptible–infected pairing, where behaviour is concerned, susceptible individuals are more likely to adopt when interacting with ingroup adopters, but less likely to adopt when interacting with outgroup adopters. We model the behavioural dynamics for members of group 1 are as follows, with analogous equations governing members of group 2:
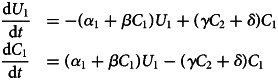


Members of group *i* may spontaneously adopt the behaviour independent of direct social influence, and do so at rate *α_i_*. This adoption may be due to individual assessment of the behaviour's utility, to influences separate from peer mixing, such as from media sources, or to socioeconomic factors that make behaviour adoption more or less easy for certain groups. For these reasons, we assume that groups can differ in their rates of spontaneous adoption. In reality, it is possible for groups to differ on all four model parameters, all of which can influence differences in adoption rates. For simplicity, we restrict our analysis to differences in spontaneous adoption.

Uncareful individuals are positively influenced to become careful by observing careful individuals of their own group, with strength *β*. However, this is countered by the force of outgroup aversion, *γ*, whereby individuals may cease being careful when they observe this behaviour among members of the outgroup. The behaviour is eventually discarded at rate *δ*, representing the financial and/or psychological costs of continuing to adopt preventive behaviours like social distancing and wearing face masks.

This model assumes no explicit homophily in terms of behavioural influence. On the one hand, it seems obvious that we observe and communicate with those in our own group more than other groups. On the other hand, opportunities for observing outgroup behaviours are abundant in a digitally connected world, which alter the conditions for cultural evolution (Acerbi, 2019). For simplicity, we do not add explicit homophily terms to this system. Instead, we simply adjust the relative strengths of ingroup influence and outgroup aversion, *β*/*γ*. When this ratio is higher, it reflects stronger homophily for behavioural influence.

**Figure 2.**
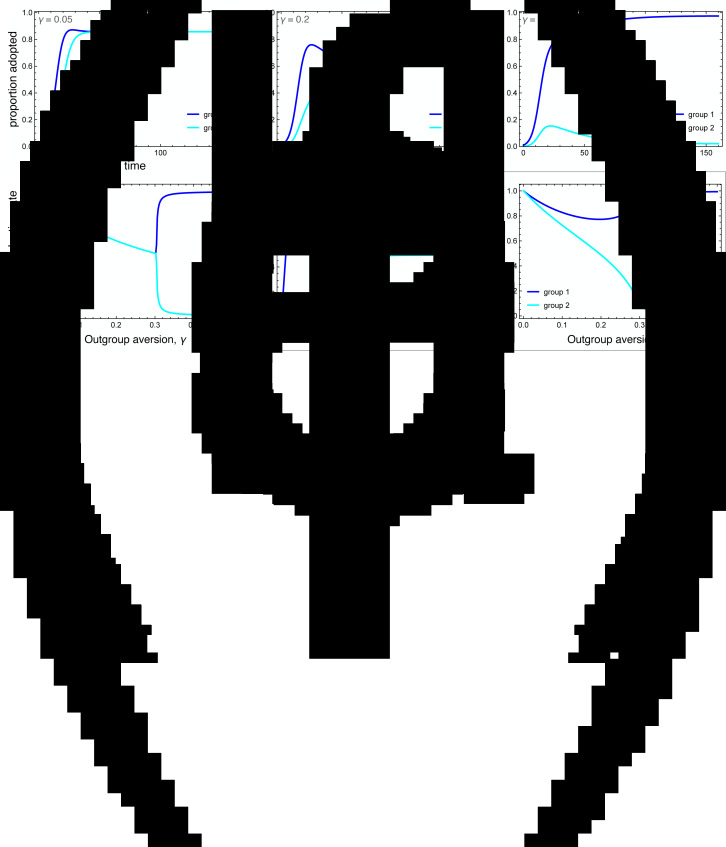
Dynamics of the behavioural adoption. (a–c) Behaviour adoption dynamics in each group for different levels of outgroup aversion, *γ*. Parameters used were *α*_1_ = *α*_2_ = 0.001, *β* = 0.3, *δ* = 0, *C*_1_(0) = 0.01, *C*_2_(0) = 0. (d) Equilibrium adoption rates for each group as a function of outgroup aversion, *γ*. A bifurcation occurs when outgroup aversion overpowers the forces of positive influence. (e) Behaviour adoption dynamics for *γ* = 0.2 where group 1 has a higher spontaneous adoption rate, *α*_1_ = 0.1. Here, the two groups converge to different equilibrium adoption rates. (f) Equilibrium adoption rates for each group as a function of outgroup aversion, *γ*, when *α*_1_ = 0.1.

Numerical simulations that illustrate the influence of outgroup aversion are depicted in Figure 2. In all cases, the careful behaviour is first adopted by group 1. In the absence of outgroup aversion, both groups adopt the behaviour at saturation levels, with group 2 being slightly delayed. When outgroup aversion is added, the delay increases, but more importantly, overall adoption declines for both groups. This decline continues as long as the strength of outgroup aversion is less than the strength of positive ingroup influence. A phase transition occurs here (Figure 2c, d). Although group 2 may initially adopt the behaviour, adoption is subsequently suppressed, resulting in a polarising behaviour that is abundant in group 1 but nearly absent in group 2.

We also consider the case in which one group has a higher intrinsic adoption rate, which could be driven by differences in personality types, norms or media exposure between the two groups. When *α*_1_ > *α*_2_, the equilibrium adoption rate for group 1 could be considerably higher than for group 2, even when ingroup positive influence was greater than outgroup aversion (Figure 2e, f). Note that these differences arise entirely because of outgroup aversion. When *γ* = 0, both groups adopt at maximum levels.

Outgroup aversion has a strong influence on adoption dynamics. It can delay adoption, reduce equilibrium adoption rates and even suppress adoption entirely in the later-adopting group. As we will see, when the behaviour being adopted influences disease transmission, quite interesting dynamics can emerge.

## 4. Coupled contagion with homophily and outgroup aversion

Before we explore the coupled dynamics of this system, we must add one more consideration to the model. We focus on the adoption of preventative behaviours that decrease the effective transmission rate of the infection, such as social distancing or wearing face masks. We model this by asserting that the transmission rate is *τ*_C_ for careful individuals and *τ*_U_ for uncareful individuals, such that *τ*_U_ ≥ *τ*_C_. When considering the interaction between careful and uncareful individuals, we use the geometric mean, so the transmissibility between SU and IU (that is, between susceptible and infected individuals who are both uncareful) is 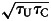. We use the geometric mean so that if either population reduces its transmissibility to zero, transmission among its members becomes impossible.

**Figure 3.**
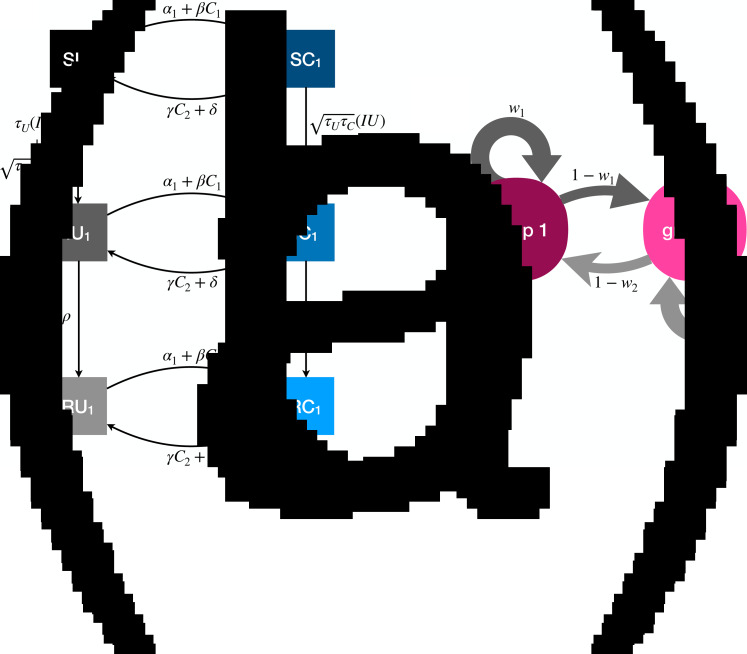
Illustration of the dynamics for the coupled contagion model. (a) Transition probabilities between compartments for members of group 1. For simplicity these probabilities do not include the influence of homophily. (b) Homophilous interactions. Members of group *i* have physical contact with members of their own group with probability *w*_*i*_ and members of the outgroup with probability 1 − *w*_*i*_.

The full model has six compartments, with two-letter abbreviations denoting the disease and behavioural state (Figure 3). The coupled dynamics for members of group 1 are as follows, with analogous equations governing members of group 2, such that the full system is defined by 12 coupled differential equations. A list of all parameters is presented in Table 1.
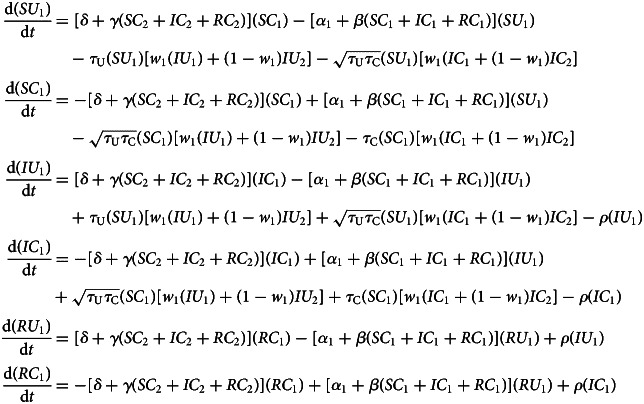

**Table 1.** Model parameters

Parameter	Definition
*τ*_C_	Disease transmissibility for careful individuals
*τ*_U_	Disease transmissibility for uncareful individuals
*ρ*	Disease recovery rate
*w*_*i*_	Homophily for group *i*
*α*_*i*_	Spontaneous behaviour adoption rate for group *i*
*β*	Ingroup positive influence on behaviour
*γ*	Outgroup negative influence on behaviour
*δ*	Behaviour discard rate

Behavioural adoption is independent of infection status in this model. This may not be a realistic assumption for some systems, such as Ebola, where the both the infection status of the adopter and the perceived incidence in the population are likely to influence behaviour. The assumption seems more realistic for infections like influenza and COVID-19, where infection status is not always transparent and decisions are likely to be made on the basis of more abstract socially-transmitted information. There are intermediate cases, however, such as where media reports of disease prevalence or the perceived availability may influence the adoption of preventative behaviours (Lau et al., 2010; Zhang et al., 2015; Seale et al., 2020). We do not consider such cases here.

To make the behavioural adoption most meaningful, we focus on the case where instantaneous and universal adoption of the careful behaviour would decrease the disease transmissibility so that *R*_0_ < 1. That is, if everyone immediately adopted the behaviour, the epidemic would fizzle out. However, behaviour adoption doesn't typically work this way. We have already noted that under assumptions of between-group variation and outgroup aversion, a behaviour is likely to be adopted neither instantaneously nor universally. The question we tackle now is how those socially driven facets of behavioural adoption influence disease dynamics.

**Figure 4.**
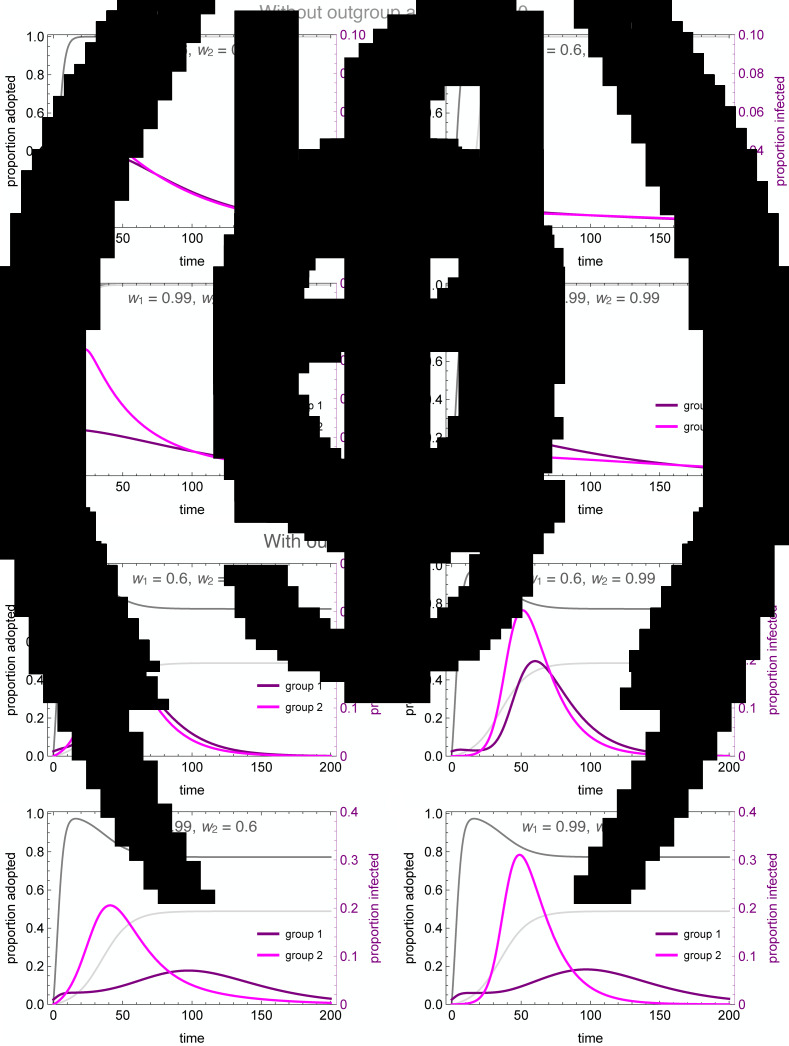
Coupled contagion dynamics when the behaviour leads to highly effective reduction in transmissibility, under varying conditions of homophily and outgroup aversion. Notice difference in *y*-axis scale for infection rate between top and bottom sets of graphs. Parameters used: *τ*_*U*_ = 0.3, *τ*_*C*_ = 0.069, *ρ* = 0.07, *α*_2_ = 0.1, *α*_2_ = 0.001, *β* = 0.3, *δ* = 0, *SU*_1_(0) = 0.98, *SC*_1_(0) = 0.01, *IU*_1_(0) = 0.01, *IC*_1_(0) = *RU*_1_(0) = *RC*_1_(0) = 0, *SU*_2_(0) = 1.0, *SC*_2_(0) = *IU*_2_(0) = *IC*_2_(0) = *RU*_2_(0) = *RC*_2_(0) = 0.

Figure 4 illustrates the wide range of possible disease dynamics under varying assumptions of homophily and outgroup aversion. A wider range of homophily values are explored in the Supplemental Materials (Figures S4 and S5). In the absence of either homophily or outgroup aversion, our results mirror previous work on coupled contagion in which the adoption of inhibitory behaviours reduces peak infection rates, flattening the curve of infection. Owing to differences in spontaneous adoption rates, however, group 2 may see a higher peak infection rate even when the infection breaks out in group 1, because the inhibitory behaviour spreads more slowly in that group (Figure 4a).

Homophilous interactions further lower infection rates. If group 1 alone is homophilous, the infection rate declines in that group, while peak infections actually increase in group 2 (Figure 4c). This is because group 1 adopts the careful behaviour early, decreasing their transmission rate and simultaneously avoiding contact with the less careful members of group 2, who become infected through their frequent contact with group 1. If group 2 alone is homophilous, on the other hand, the infection is staved off even more so than if both groups are homophilous (Figure 4b, d). This is because members of group 2 avoid contact with group 1 until the careful behaviour has been widely adopted, while members of group 1 diffuse their interactions with some members of group 2, and these are less likely to lead to new infections.

Outgroup aversion considerably changes these dynamics. First and foremost, outgroup aversion leads to less widespread adoption of careful behaviours, dramatically increasing the size of the epidemic. Moreover, because under many circumstances there will be between-group differences in equilibrium behaviour-adoption rates, this can lead to dramatic group differences in infection dynamics. In the absence of outgroup aversion, we saw that homophily in group 2 could lead to an almost total suppression of the epidemic. Not so with outgroup aversion, in which the peak infection rates *increase* relative to the low-homophily case (Figure 4e, f). This occurs because homophily causes a delay in the infection onset in group 2. Behavioural adoption slows the epidemic initially in both groups. However, when the infection finally reaches group 1, behavioural adoption has decreased past its maximum owing to the outgroup aversion, causing peak infections in both groups to soar.

The dynamics are particularly interesting for the case where the group in which the epidemic first breaks out (group 1 in our analyses) is also strongly homophilous. Owing to homophily along with rapid behaviour adoption, the epidemic is initially suppressed in this group. However, owing to slower and incomplete behaviour adoption, the infection spreads rapidly in group 2. As the infection peaks in group 2 while group 1 decreases its behaviour adoption rate, we observe a delayed ‘second wave’ of infection in group 1, well after the infection has peaked in group 2 (Figure 4g). This effect is exacerbated when both groups are homophilous, as the epidemic runs rampant in the less careful group 2 (Figure 4h). As shown in the Supplementary Material, the timing of the second wave is also delayed to a greater extent when the adopted behaviour is more efficacious at reducing transmission (Figure S6).

**Figure 5.**
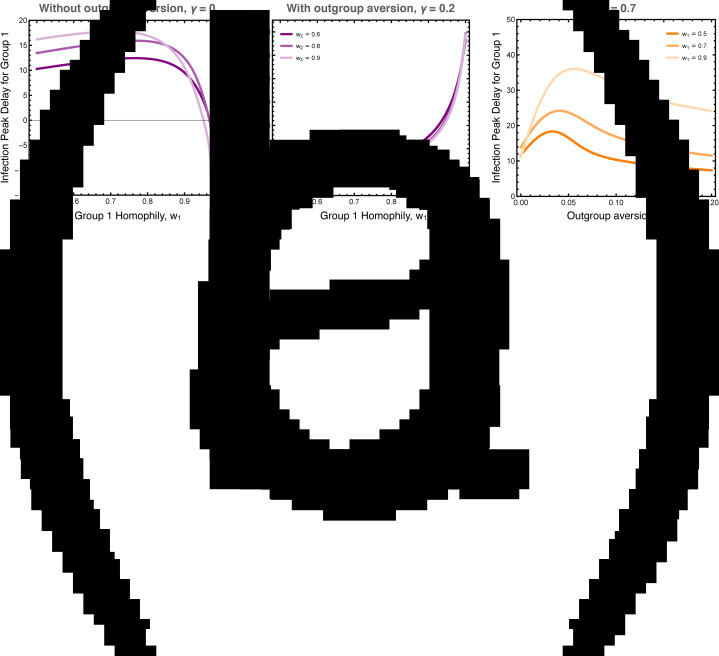
Difference in the timing of the peak infection rates between groups. These plots show the extend to which the peak in group 1 lags behind the peak in group 2. The first two plots show the peak delay for group 1 as a function of group 1 homophily, (a) with and (b) without outgroup aversion, *γ*. The third plot (c) more systematically varies outgroup aversion, for several values of group 1 homophily and moderate group 2 homophily, *w*_2_ = 0.7. Other parameters used: *τ*_*U*_ = 0.3, *τ*_*C*_ = 0.069, *ρ* = 0.07, *α*_2_ = 0.1, *α*_2_ = 0.001, *β* = 0.3, *δ* = 0.

We explored the differences in the timing of the infection peaks between the two groups, as illustrated in Figure 5. As noted, homophily in group 1 has a larger effect than homophily in group 2 because the infection first breaks out in group 1. Without outgroup aversion, the infection peak in group 1 is usually closely timed to the infection peak in group 2, usually coming slightly later owingto group 2's lagged adoption of the preventative behaviour (Figure 5a). If group 1 has very strong homophily, however, the infection can peak earlier there, as its spread to group 2 is impeded. When outgroup aversion is strong, however, group 2's adoption of the preventative behaviour is severely impeded, which causes its infection rate to peak much earlier than in group 1, and this effect is only bolstered by strong homophily in group 1 (Figure 5b). The effect of outgroup aversion on the differential timing between groups of infection rate peaks is non-monotonic (Figure 5c), peaking at intermediate values of *γ*.

## 5. Discussion

It is well known that disease transmission is influenced by behaviour. What is often overlooked is how behaviour itself changes within heterogeneous cultural populations. Both population structure and social identity influence who interacts with whom, affecting disease transmission, and who learns from whom, affecting behaviour change. We have highlighted two of these forces – homophily and outgroup aversion – and shown their dramatic influence on disease dynamics in a simple model.

In terms of social interaction and behaviour adoption dynamics, group identity exerts its influence by way of homophily, a powerful social force. Aral et al. (2009), for example, showed that homophily accounted for more than 50% of contagion in a natural experiment on behavioural adoption. The effect of homophily on diffusion dynamics can be variable. For example, homophily can slow down convergence towards best responses in strategic networks (Golub & Jackson, 2012). This can be critical when the time scales of learning and infection are different. Homophily can also lower the threshold for desirability (or the selective advantage) required for adoption of a behaviour. Creanza and Feldman (2014) showed that homophily and selection can have balancing effects – the selective advantage of a trait doesn't need to be as high to spread when it is transmitted assortatively by its bearers. In the case of our coupled-contagion model, strong homophily interferes with the adaptive adoption of protective behaviour. Centola (2011) showed that homophily can increase the rate of adoption of health behaviours, but his experimental population could assort only on positive cues, and had no ability to signal or perceive group identity.

Consider the observed adoption dynamics under differential homophily. When the homophily of group 1 is less than that of group 2, group 1 can be interpreted as ‘frontline’ workers, who are exposed to a broader cross-section of the population by nature of their work. Outgroup avoidance of this group's adopted protective behaviour can arise if there are status differentials across the groups. Prestige bias, the tendency to adopt behaviours associated with high-status individuals, is a mechanism that can drive differential uptake of novel behaviour by different groups (Boyd & Richerson, 1985), for which there is quite broad support (Jiménez & Mesoudi, 2019). When both groups are highly homophilous and outgroup aversion is strong, the resulting dynamics suggest the case of negative partisanship, a type of outgroup aversion in which partisans select actions based not on explicit policy preferences but in opposition to the outgroup (Abramowitz & Webster, 2016). In this case, differences in the relative size of the epidemic will be driven purely by differences in the rates of preventative behaviour adoption by the two groups, including those differences induced by outgroup aversion.

Incorporating adaptive behaviour into epidemic models has been shown to significantly alter dynamics (Fenichel et al., 2011). Prevalence-elastic behaviours (Funk et al., 2010) are those that increase with the growth of an epidemic. While these behaviours may be protective, they can also lead to cycling of incidence, which can prolong epidemics. Similarly, the adoption of some putatively protective behaviours that are actually ineffective can be driven by the existence of an epidemic when the cost of adoption is sufficiently low (Tanaka et al., 2009). We have shown in this paper that group-identity processes can have large effects, leading groups that would otherwise respond adaptively to the threat of an epidemic to behave in ways that put them, and the broader populations in which they are embedded, at risk.

The context of the ongoing COVID-19 pandemic provides some interesting and timely perspective on the relationship between behaviour, adaptive or otherwise, and transmission dynamics. While there remains much uncertainty about the infection fatality ratio of COVID-19, and how this varies according to individual, social and environmental context, it is clear that the great majority of infections do not lead to death (Russell et al., 2020; Meyerowitz-Katz & Merone, 2020). Furthermore, the extensive presymptomatic (or even asymptomatic) transmission of the SARS-CoV-2 (He et al., 2020; Li et al., 2020; Arons et al., 2020) is likely to reduce associations between behaviour and local infection rates. We expect that such a situation will not induce strong prevalence-elastic behavioural responses, and that the sorts of identity-based responses we describe here will dominate the behavioural effects on transmission.

How do we intervene in a way to offset the pernicious effects of negative partisanship on the adoption of adaptive behaviour? While it may seem obvious, strategies for spreading efficacious protective behaviours in a highly structured population with strong outgroup aversion will require weakening of the association between protective behaviours and particular subgroups of the population. Given that we are writing this during a global pandemic in which perceptions and behaviours are highly polarised along partisan lines, attempts to mitigate partisanship in adaptive behavioural responses seem paramount to support.

The models we have analysed in this paper are broad simplifications of the coupled dynamics of behaviour-change and infection. It would therefore be imprudent to use them to make specific predictions. The goal of this approach is to develop strategic models in the sense of Holling (1966), sacrificing precision and some realism for general understanding of the potential interactions between social structure, outgroup aversion and coupled contagion (Levins, 1966; Smaldino, 2017). Such models provide a scaffold for the development of richer theories concerning coupled disease and behavioural contagions.

## Supplementary material

For supplementary material accompanying this paper visit https://doi.org/10.1017/ehs.2021.22. click here to view supplementary material
