# Supplementary material for: Coupled dynamics of behaviour and disease contagion among antagonistic groups
Source: Evol Hum Sci. 2021 Mar 18;3:e28. doi: 10.1017/ehs.2021.22 (PMC10427326; doi:10.1017/ehs.2021.22)
Supplement: Supplementary file 1 [file S2513843X21000220sup001.pdf]

APPENDIX (ONLINE SUPPLEMENT)
Coupled Dynamics of Behavior and Disease Contagion Among Antagonistic Groups

PAUL E. SMALDINO[1,*] AND JAMES HOLLAND JONES[2]

Appendix A. The SIR model with homophily

We extended the SIR model to explore scenarios where individuals assort based on identity, as described in the main text. Here we present some additional analyses of this model.

Figure S1 illustrates that when an infection breaks out in group 1, homophily can delay the outbreak of the epidemic in group 2. Homophily for each group works somewhat synergistically, but the effect is dominated by w_2. This is because the infection spreads rapidly in a homophilous group 1, and if group 2 is not homophilous its members will rapidly become infected. However, if group 2 is homophilous, its members can avoid the infection for longer, particularly when group 1 is also homophilous.

We also explored a scenario where R_0 for the basic model was very close to 1, indicating a small epidemic (we used $R_0 = 1.14$; Figure S2). Note that this calculation of R_0 does not account for homophily; we derive R_0 for the homophily model in the SI Appendix and show that this is a reasonable approximation. When homophily was low ($w = 0.6$), the populations mixed a lot. The proportion of infected individuals in group 1 briefly fell, as the majority of new infected individuals were in group 2. However, the groups quickly matched their pace and experienced the outbreak in tandem. When homophily was high ($w = 0.99$), not only did group 2 experience a delayed outbreak, it also experienced a substantially lower peak infection rate, because the total number of infected individuals at the start of its outbreak was so much lower than that experienced by group 1. Thus, homophily can serve not only to delay an epidemic, but also to reduce it in the cases of lower transmissibility infections.

Appendix B. Basic Reproduction Number

We can calculate the basic reproduction number, R_0, for the homophily model. We employ the next-generation matrix approach described by Heffernan et al. (2005), which concisely summarizes the ideas for calculating R_0 in structured populations articulated by, e.g., Diekmann et al. (1990) and van den Driessche and Watmough (2002).

[1]University of California, Merced, USA
[2]Stanford University, USA
E-mail address: paul.smaldino@ucmerced.edu.

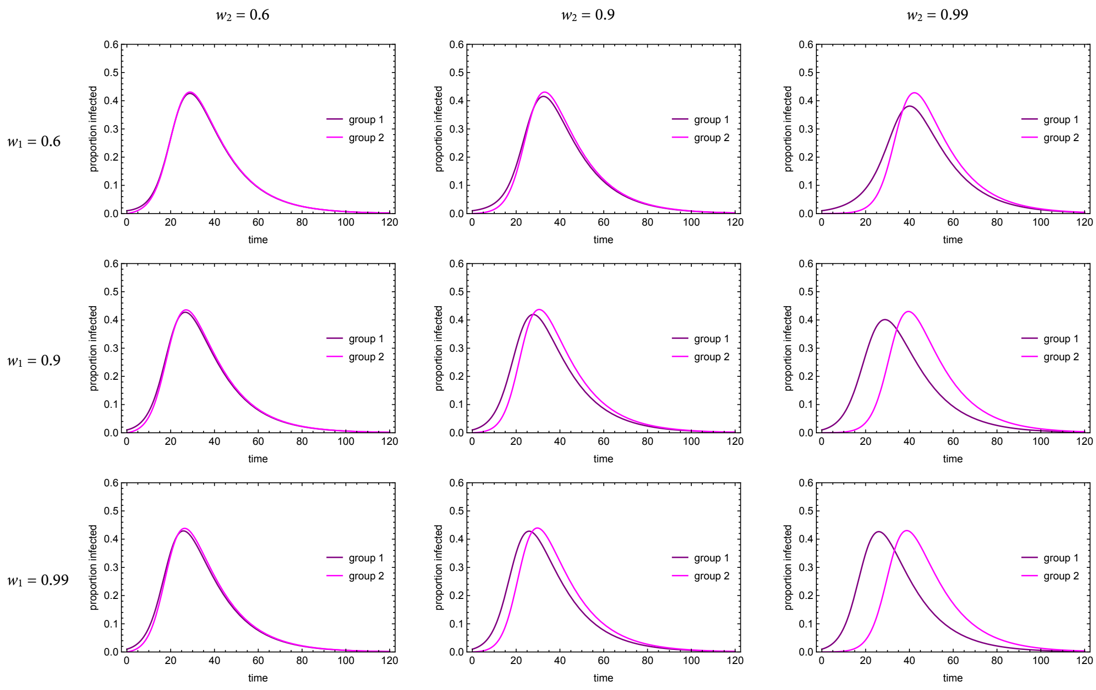

FIGURE S1. Infection dynamics in the SIR model with asymmetric homophily. Here $\tau = 0.3$, $\rho = 0.07$.

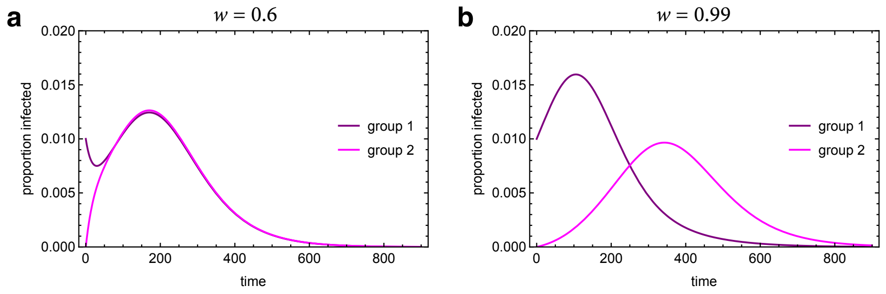

FIGURE S2. Infection dynamics in the SIR model with homophily when $R_)$ is close to 1. Here $\tau = 0.08$, $\rho = 0.07$, $w_1 = w_2 = w$.

Following the notation of Heffernan et al. (2005), the next generation matrix $\mathbf{G}$ is comprised of two component matrices: $\boldsymbol{F}$ and $\boldsymbol{V}^{-1}$, where

$$\boldsymbol{F} = \left[\frac{\partial F_i(x_0)}{\partial x_j}\right], \tag{1}$$

and

$$\boldsymbol{V} = \left[\frac{\partial V_i(x_0)}{\partial x_j}\right]. \tag{2}$$

These are square matrices of the partial derivatives of new infections (F_i) and transfers between different compartments (V_i). The rank of these matrices is the number of distinct classes of infections. x_0 is the disease-free equilibrium state. This matrix should be non-negative, irreducible, and primitive.

is given by the dominant eigenvalue of the matrix $\boldsymbol{G} = \boldsymbol{F}\boldsymbol{V}^{-1}$.

For the homophily model, the only two equations that yield new infections are those for $\dot{I}_1$ and $\dot{I}_2$:

$$\frac{dI_1}{dt} = \tau S_1\left(w_1 I_1 + (1-w_1)I_2\right) - \rho I_1 \tag{3}$$

$$\frac{dI_2}{dt} = \tau S_2\left(w_2 I_2 + (1-w_2)I_1\right) - \rho I_2 \tag{4}$$

Applying the next-generation-matrix approach described above to these equations, and noting that in the disease-free equilibrium $S_1 + S_2 = 1$, we get the next-generation matrix:

$$\boldsymbol{G} = \begin{pmatrix} \frac{S_1 \tau w_1}{\rho} & \frac{S_1 \tau (1-w_1)}{\rho} \\ \frac{S_2 \tau (1-w_2)}{\rho} & \frac{S_2 \tau w_2}{\rho} \end{pmatrix}. \tag{5}$$

Letting $S_2 = 1 - S_1$ in the disease-free equilibrium, the larger of the two eigenvalues of this matrix is:

$$R_0 = \frac{\tau}{2\rho}\left(\sqrt{S_1^2 w_1^2 + 2(1-S_1)S_1 w_1 w_2 - 4(1-S_1)S_1 w_1 + (1-S_1)^2 w_2^2 - 4(1-S_1)S_1 w_2 + 4(1-S_1)S_1 + \rho^2 S_1 w_1 + \rho^2 (1-S_1) w_2}\right) \tag{6}$$

This relationship is greatly simplified by assuming uniform homophily ($w_1 = w_2 = w$):

$$R_0 = \frac{\tau}{2\rho}(w + \sqrt{S_1^2(8w-4) + S_1(4-8w) + w^2}). \tag{7}$$

Note that if we collapse the structure of the population such that $S_1 = 1$ (which also implies that $w = 1$), then equation 7 reduces to $R_0 = \tau/\rho$, the standard definition for the basic reproduction number in an unstructured SIR model (Keeling and Rohani, 2007).

We see from figure S3 that structure and homophily (in the absence of coupled adaptive behavior and outgroup aversion) are actually somewhat protective from an epidemic perspective. R_0 is lowest when the population is evenly split between the two groups and when homophily is extreme. This makes sense since structure generally slows epidemics by subdividing the potential for contacts and thereby slowing mixing (Arthur et al., 2017).

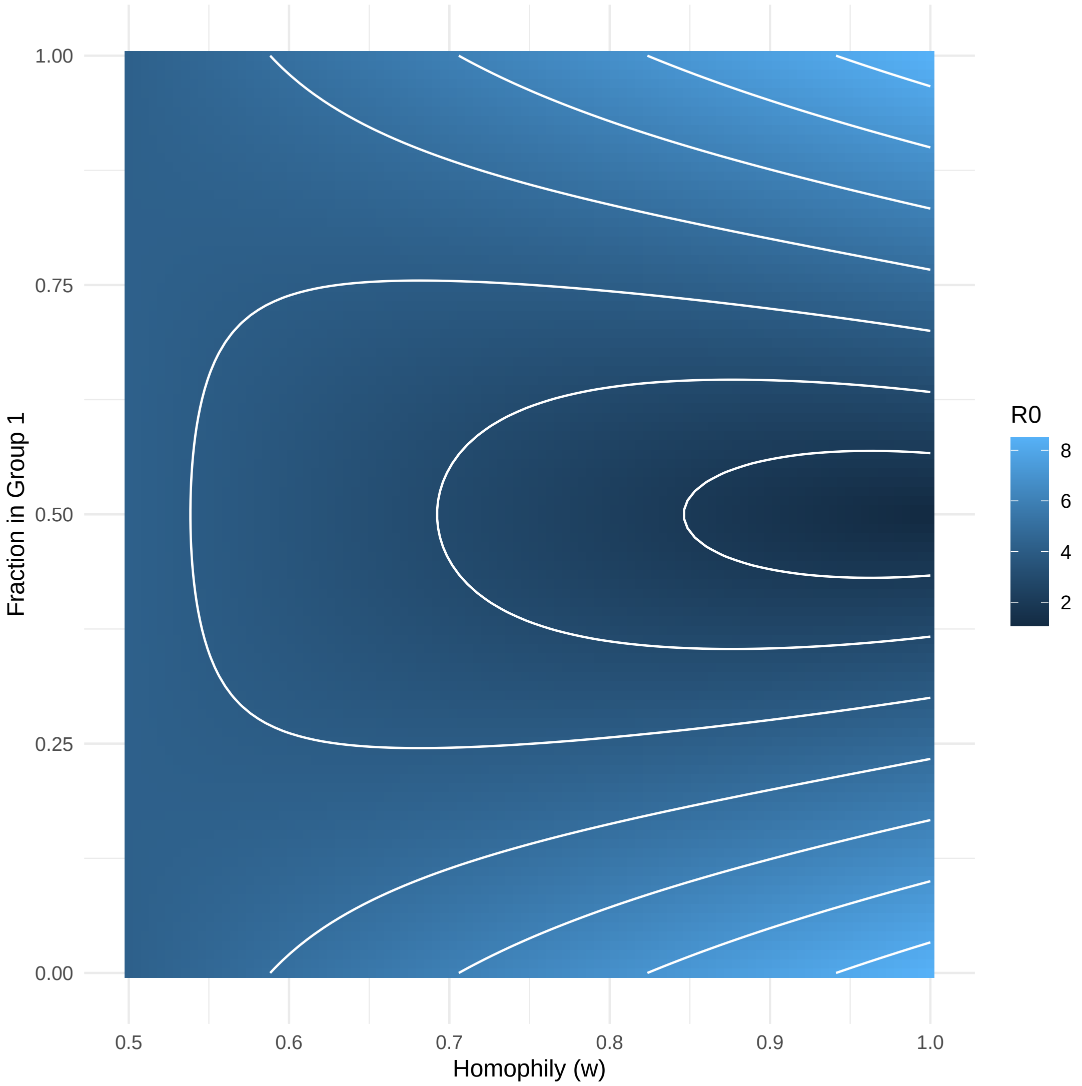

FIGURE S3. R_0 for the uniform-homophily model (Equation 7) as a function of the strength of homophily (w) and the initial population structure. $\tau = 0.3$, $\rho = 0.07$.

APPENDIX C. COUPLED CONTAGION DYNAMICS

Here we present an extended version of the full model analysis presented in the main text, that includes intermediate homophily of $w_i = 0.9$. Analysis with no outgroup aversion is shown in Figure S4, and with outgroup aversion is shown in Figure S5. The figures illustrate how homophily and outgroup aversion can interact to produce unintuitive dynamics. When both forces are present, an infection that begins in group 1

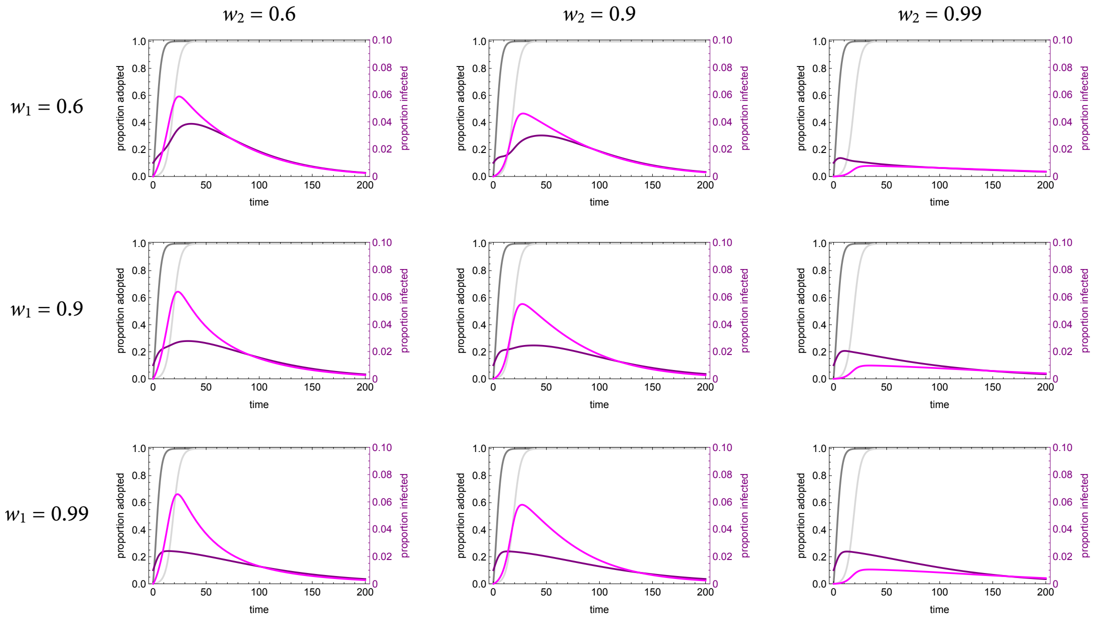

Figure S4. Coupled dynamics of the full model without outgroup aversion ($\gamma = 0$) for with varying homophily. Darker lines are group 1, lighter lines are group 2. Parameters used: $\tau_U = 0.3$, $\tau_C = 0.069$, $\rho = 0.07$, $\alpha_2 = 0.1$, $\alpha_2 = 0.001$, $\beta = 0.3$, $\delta = 0$.

can peak earlier and stronger in group 2, followed by a smaller peak in the group where it began.

Appendix D. Analysis of behavioral efficacy

In the main text analysis, we assumed that the adopted behavior reduced the transmission to below the threshold for $R_0 < 1$. In other words, if everyone immediately and universally adopted the behavior at the start of the outbreak, it would not become an epidemic. Although we view this as a reasonable assumption (that is, the efficacy of the behavior is reasonable, not the expectation that it will be either immediately or universally adopted), it is also worth examining what happens with the spread of behaviors that reduce transmission, but not below epidemic levels. Figure S6 illustrates the model dynamics for varying levels of behavior efficacy (τ_C) with and without outgroup aversion and for both weak and strong homophily.

Without outgroup aversion ($\gamma = 0$), the effect is clear: the more efficacious the behavior, the smaller the epidemic. This occurs because the behavior spreads effectively. With outgroup aversion, two things happen. First, the more effectively the behavior reduces transmission (that is, the smaller τ_C is), the smaller the overall epidemic, but with an effect that is much stronger in group 1. In group 2, the effect of increased behavior efficacy is relatively small, because adoption is reduced and delayed. Second, the better the behavior reduces transmission, the bigger the delay in when group 1 experiences a

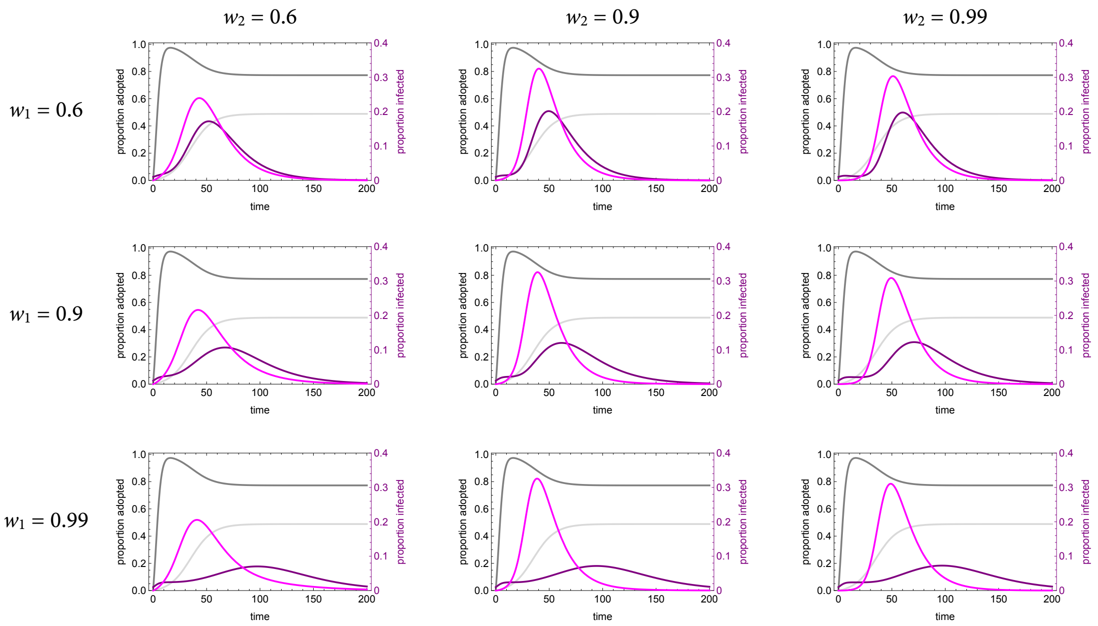

FIGURE S5. Coupled dynamics of the full model with outgroup aversion ($\gamma = 0.2$) for with varying homophily. Darker lines are group 1, lighter lines are group 2. Parameters used: $\tau_U = 0.3$, $\tau_C = 0.069$, $\rho = 0.07$, $\alpha_2 = 0.1$, $\alpha_2 = 0.001$, $\beta = 0.3$, $\delta = 0$.

"second wave." This illustrates that the dynamics of disease transmission can become quite complex when even simple assumptions about behavior and group structure are considered.

References

Arthur, R. F., Gurley, E. S., Salje, H., Bloomfield, L. S. P., and Jones, J. H. (2017). Contact structure, mobility, environmental impact and behaviour: the importance of social forces to infectious disease dynamics and disease ecology. *Philosophical Transactions of the Royal Society B: Biological Sciences*, 372(1719):1–9.

Diekmann, O., Heesterbeek, J. A. P., and Metz, J. A. J. (1990). On the definition and the computation of the basic reproduction ratio r0 in models for infectious diseases in heterogeneous populations. *Journal of Mathematical Biology*, 28(4):365–382.

Heffernan, J., Smith, R., and Wahl, L. (2005). Perspectives on the basic reproduction ratio. *Journal of the Royal Society Interface*, 2:281–293.

Keeling, M. J. and Rohani, P. (2007). *Modeling Infectious Diseases in Humans and Animals*. Princeton University Press, Princeton.

van den Driessche, P. and Watmough, J. (2002). Reproduction numbers and subthreshold endemic equilibria for compartmental models of disease transmission. *Mathematical Biosciences*, 180:29–48.

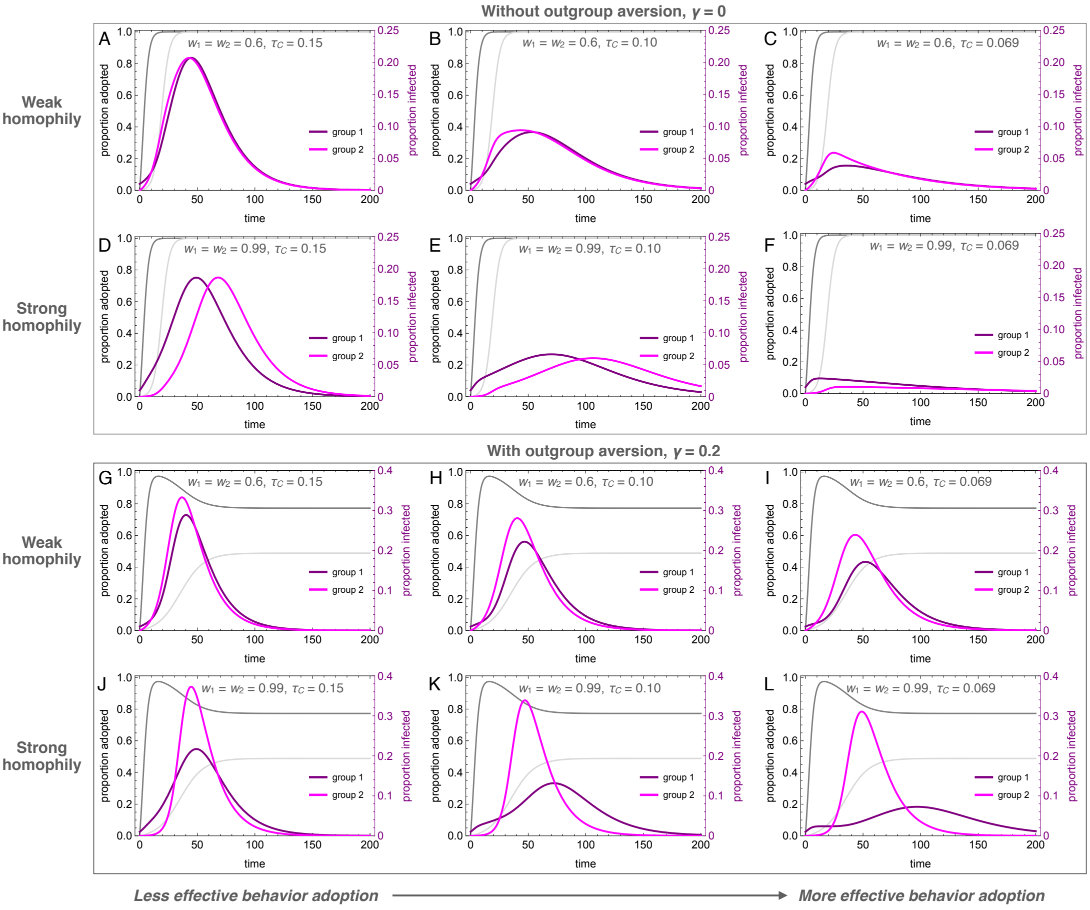

Figure S6. Coupled dynamics of the full model for varying levels of behavior efficacy, $\tau_C = \{0.15, 0.1, 0.069\}$, where only the last case would provide $R_0 < 1$ if immediately and universally adopted at the start of the outbreak. We provide analyses with and without outgroup aversion and for both weak and strong homophily. Darker lines are group 1, lighter lines are group 2. Parameters used: $\tau_U = 0.3$, $\rho = 0.07$, $\alpha_2 = 0.1$, $\alpha_2 = 0.001$, $\beta = 0.3$, $\delta = 0$.
